# Anti-*Ascaris* IgG Seroprevalence and ELISA Performance Among Adults in Three Regions of Kazakhstan

**DOI:** 10.3390/life16101635

**Published:** 2026-09-30

**Authors:** Berik M. Khairullin, Mukhit B. Orynbayev, Timur I. Davlyatshin, Kulyaisan T. Sultankulova, Aiganym A. Tussipova, Sabyrkhan M. Barmak, Asylzhan A. Myrzakhmet, Oralkhan Tusipkanuly, Dulat A. Inkarbekov, Timur T. Davlyatshin, Andrey M. Dmitrovskiy, Sandugash E. Mukazhanova, Gaukhar E. Nusupbaeva, Aidar M. Namet

**Affiliations:** 1 Scientific Research and Production Center «MVA GROUP» LLP, 1124, Accounting Quarter 096, Koksay Village, Irgeli Rural District, Karasay District, Almaty 040921, Kazakhstan; 2 LLP «Research Institute for Biological Safety Problems» JSC «QazBioPharm» of the Ministry of Health of the Republic of Kazakhstan, Gvardeysky 080409, Kazakhstan; 3 National Center for Biotechnology, Central Reference Laboratory, 14 Zhahanger St., Almaty 050054, Kazakhstan; 4 RSE «Center for Sanitary and Epidemiological Expertise of the Medical Center of the Presidential Administration of the Republic of Kazakhstan», Astana Z19Y8B0, Kazakhstan

**Keywords:** ascariasis, *Ascaris lumbricoides*, seroprevalence, ELISA, Kato–Katz, adults, Kazakhstan

## Abstract

Regional surveys can help characterize adult exposure to *Ascaris* and assess the interpretation of different laboratory markers. This cross-sectional study examined 2001 volunteers from the Akmola, Pavlodar, and North Kazakhstan Regions during 2025–2026. Questionnaire data were collected, and paired serum and stool specimens were tested using the MVA GROUP IgG ELISA and Kato–Katz method. Associations with seropositivity were evaluated using multivariable logistic regression, and 300 selected sera were compared with the DRG *Ascaris* IgG ELISA. Overall seroprevalence was 28.0% (95% CI: 26.1–30.0%), with regional differences remaining after adjustment for age, sex, residence, and sampling year. Kato–Katz positivity was 8.4%, without a significant regional difference. ELISA sensitivity and specificity relative to Kato–Katz were 85.2% and 77.2%, respectively. Agreement with DRG was 95.0% (κ = 0.900). These findings support further regional assessment of adult exposure. Agreement between ELISAs supports consistent antibody classification in the selected panel, while discordance with Kato–Katz requires separate interpretation of antibody detection and intestinal egg shedding.

## 1. Introduction

Ascariasis is a soil-transmitted helminth infection caused predominantly by *Ascaris lumbricoides*. The World Health Organization (WHO) reports that soil-transmitted helminth infections collectively affect approximately 1.5 billion people worldwide [1], while the US Centers for Disease Control and Prevention (CDC) estimates that 772–892 million people are infected with *Ascaris* [2]. Although the age-standardized prevalence of ascariasis declined by an estimated 75.8% between 1990 and 2021, the burden remains unevenly distributed and is highest in populations with lower socioeconomic development [3]. In 2024, more than 883 million children required preventive chemotherapy for soil-transmitted helminthiases, but only 506.6 million received treatment, corresponding to 57.4% global coverage [4]. Thus, despite substantial progress, incomplete treatment coverage and continued environmental exposure allow transmission to persist.

This persistence is closely related to the life cycle of the parasite. Infection occurs through ingestion of embryonated eggs from fecally contaminated soil, water, food, or hands. Eggs passed in feces must mature in the environment before becoming infective; consequently, ascariasis is not transmitted directly from person to person [1,2]. After ingestion, the larvae migrate through the liver and lungs before returning to the small intestine, where adult worms develop. Most light infections are asymptomatic, whereas heavier infections may cause abdominal symptoms, impaired growth in children, intestinal obstruction, or migration of adult worms into the biliary and pancreatic ducts [2,5]. Because the eggs can survive in soil for prolonged periods, infected individuals contribute to environmental contamination that may continue to expose the wider community [5].

Accordingly, the risk of infection depends not only on individual behavior but also on environmental and sanitary conditions. *Ascaris* eggs have been detected in soils and fecal sludge from urban communities with relatively high sanitation coverage, demonstrating that transmission is not confined to rural settings [6]. Vegetables and fruits may also become contaminated during cultivation, irrigation, transport, or handling. A systematic review covering 32 countries identified *A. lumbricoides* as one of the most frequently detected helminths on fresh produce [7]. Therefore, sanitation, water supply, soil contact, and food-handling practices should be evaluated as separate exposure factors rather than inferred solely from urban or rural residence. These links are relevant to a One Health approach, which connects human, animal, and environmental health through coordinated disease prevention and surveillance [8]. Adult surveys can provide human exposure data to guide investigations of environmental contamination and locally relevant transmission pathways.

In addition to these epidemiological differences, the measured prevalence of ascariasis depends on the diagnostic method used. The Kato–Katz method remains the WHO-recommended reference copromicroscopic approach for detecting soil-transmitted helminth eggs. It provides direct evidence of egg shedding and permits estimation of infection intensity, although detection may be affected by low egg output and examination of a single stool specimen [9]. Serological methods provide complementary information because they detect the host immune response to parasite antigens. Anti-*Ascaris* IgG may indicate previous or current immunological exposure, including in individuals without detectable egg shedding. However, antibodies may persist after infection and may react with antigens of related helminths; therefore, seropositivity alone does not confirm an active intestinal infection [10]. Copromicroscopy and serology thus describe related but distinct aspects of *Ascaris* infection and exposure.

This distinction is particularly relevant in Kazakhstan, where information on anti-*Ascaris* IgG seroprevalence among adults remains limited. Our previous study examined the relationship between serological and copromicroscopic findings in Almaty and the Almaty Region and described the application of the MVA GROUP ELISA [11]. Building on that work, the present study included 2001 adults from the Akmola, Pavlodar, and North Kazakhstan Regions. It aimed to determine regional anti-*Ascaris* IgG seroprevalence, examine demographic and questionnaire variables, evaluate the diagnostic performance of the MVA GROUP ELISA using Kato–Katz copromicroscopy as the reference method, and assess agreement with the commercial DRG ELISA.

## 2. Materials and Methods

### 2.1. Study Setting

**Figure 1:**
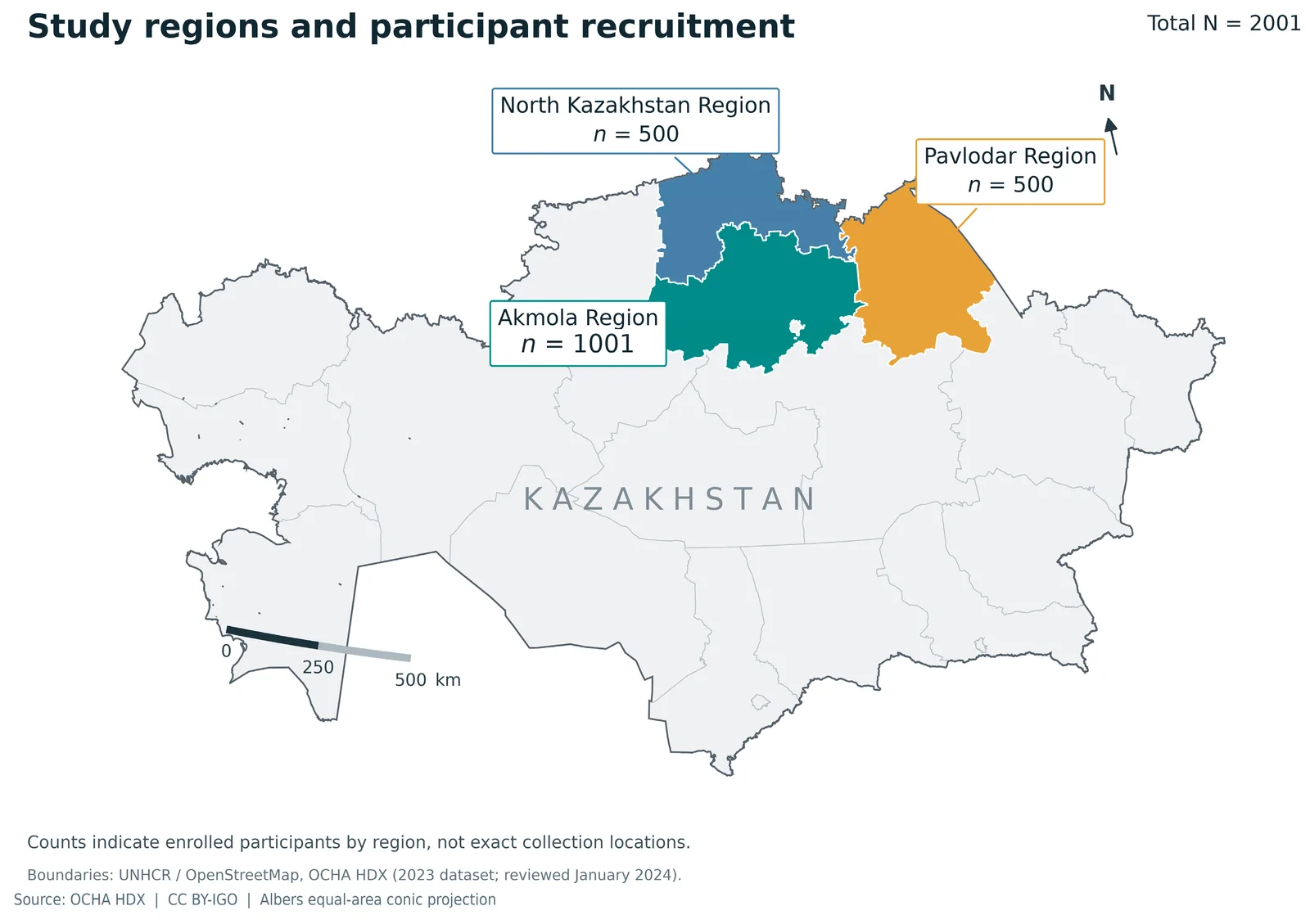
Geographical distribution of the study regions and participant recruitment in Kazakhstan. Akmola Region, *n* = 1001; Pavlodar Region, *n* = 500; North Kazakhstan Region, *n* = 500. Administrative boundaries: UNHCR/OpenStreetMap, distributed through OCHA HDX (https://data.humdata.org/dataset/cod-ab-kaz; accessed on 7 September 2026) [12].

A multiregional cross-sectional seroepidemiological study was conducted from January 2025 to May 2026 in the Akmola, Pavlodar, and North Kazakhstan Regions of Kazakhstan. The study included participants from urban and rural settlements and was designed primarily to estimate regional anti-*Ascaris lumbricoides* IgG seroprevalence and examine demographic, household, and behavioral factors associated with seropositivity. Paired copromicroscopic findings were used to evaluate the performance of the serological assay for detecting active egg-shedding infection. The study regions and participant recruitment totals are shown in Figure 1.

### 2.2. Study Population and Eligibility

Adult volunteers were recruited by non-probability sampling at the participating collection sites. A physician assessed eligibility through an interview, and volunteers received an information sheet before providing written informed consent. Eligible participants were aged ≥18 years, resided in one of the study regions, completed the study questionnaire, and provided paired venous blood and stool specimens suitable for laboratory examination. Participants who had received anthelmintic treatment during the preceding 3 months were not eligible. No restrictions were imposed according to sex or ethnicity.

The sample size reflected the monitoring program’s operational recruitment target of approximately 500 volunteers per regional sampling round. This analysis combined two rounds from Akmola (2025 and 2026), one from Pavlodar (2025), and one from North Kazakhstan (2026). The final analytical sample comprised 2001 participants: 1001 from Akmola and 500 from each of the other two regions. These totals describe completed volunteer enrollment; the estimates were not weighted to represent regional populations. Age was recorded in completed years and categorized as 18–24, 25–44, 45–59, 60–74, and ≥75 years.

### 2.3. Ethical Standards

The study was conducted in accordance with the Declaration of Helsinki [13] and national requirements for biomedical research involving human participants [14]. The protocol was approved by the Ethics Committee of the Almaty Association of Bioethics and Medical Law (Meeting No. 20, 8 January 2025).

Before enrollment, participants received information about the study procedures, specimen collection, confidentiality, and the voluntary nature of participation. Written informed consent was obtained before any study procedure was performed. Questionnaire and laboratory data were linked using unique study identification numbers and analyzed in coded form.

### 2.4. Questionnaire and Specimen Collection

Before specimen collection, participants completed a brief study-specific questionnaire with the assistance of study personnel. Questions covered age, sex, region, urban or rural residence, household conditions, ownership of dogs, cats, and domestic farm animals, drinking-water sources, access to a garden, vegetable garden, or household land plot, and dietary practices, including consumption of unwashed produce and raw or undercooked meat.

The questionnaire was used within a broader parasitological monitoring program. Animal ownership and dietary practices were examined as exploratory variables rather than assumed transmission routes for intestinal ascariasis. For analysis, animal categories were coded separately and could overlap. Marked checkbox entries were coded as reported and unmarked entries as not reported. Dietary variables represented recorded consumption of unwashed vegetables and raw meat; these were extracted from dietary text in Akmola and Pavlodar and from dedicated fields in North Kazakhstan. Blank dietary entries and missing or conflicting residence entries were treated as missing for the relevant analyses. Responses were self-reported and were not independently verified.

All participants provided paired venous blood and stool specimens. Venous blood was collected into AVATUBE^®^ vacuum serum tubes with a clot activator (Almaty, Kazakhstan). After clot formation, the samples were centrifuged at 1500–2000× *g* for 10 min. Serum was separated, transferred to labeled tubes, and stored at −20 °C until analysis.

Stool specimens were collected in sterile, leak-proof, disposable containers. Participants provided the specimens on the day of enrollment or the following day. The serum sample, stool specimen, and questionnaire belonging to each participant were labeled with the same study identification number.

### 2.5. Copromicroscopic Examination

Stool specimens were examined using the Kato–Katz thick-smear technique in accordance with World Health Organization recommendations [15]. Prepared smears were examined by light microscopy for helminth eggs. The prespecified copromicroscopic outcome for the present study was the detection of eggs morphologically compatible with *A. lumbricoides*. A specimen was classified as positive when at least 1 compatible egg was detected and negative when no compatible eggs were observed. Kato–Katz copromicroscopy was used as the reference method for detecting active intestinal infection accompanied by egg shedding.

### 2.6. Anti-Ascaris IgG ELISA

Serum specimens were tested using the Ascarida-IgG-Human-ELISA–MVA GROUP test system (Scientific Research and Production Center “MVA GROUP” LLP, Almaty, Kazakhstan), an indirect ELISA based on native excretory–secretory antigen of *A. lumbricoides*. The complete assay procedure and quality-control requirements have been described previously [11].

Briefly, serum specimens were tested in duplicate at a dilution of 1:5. Bound antibodies were detected using horseradish peroxidase-conjugated anti-human IgG, and optical density was measured at 450 nm with a reference wavelength of 630 nm. The run-specific critical optical density was calculated as

ODcrit = mean OD of the negative control + 0.200.

A specimen was classified as positive when its mean optical density was ≥ODcrit and as non-positive when its mean optical density was <ODcrit. Anti-*Ascaris* IgG positivity was interpreted as evidence of previous or ongoing immunological exposure and not, by itself, as confirmation of active intestinal ascariasis.

### 2.7. Diagnostic Performance of the MVA Group ELISA Compared with Kato–Katz Copromicroscopy

Diagnostic performance was evaluated using the paired ELISA and Kato–Katz results available for all 2001 participants. Kato–Katz copromicroscopy was defined a priori as the reference method. Results were cross-classified in a 2 × 2 table as true positive (TP), false negative (FN), false positive (FP), or true negative (TN) relative to the copromicroscopic result.

The following measures were calculated:

1. Sensitivity = TP/(TP + FN) × 100%
2. Specificity = TN/(TN + FP) × 100%
3. Positive predictive value = TP/(TP + FP) × 100%
4. Negative predictive value = TN/(TN + FN) × 100%
5. Overall accuracy = (TP + TN)/N × 100%

Agreement beyond chance was assessed using Cohen’s κ. Diagnostic-performance proportions were reported with Wilson 95% confidence intervals. These estimates describe ELISA classification relative to Kato–Katz detection of egg-shedding infection; an ELISA-positive/Kato–Katz-negative result does not, by itself, establish that the antibody result is biologically false positive.

### 2.8. Comparative Evaluation with the Commercial DRG Ascaris IgG ELISA

Following initial testing with the MVA GROUP ELISA, 300 serum specimens were selected for comparison with the commercial *Ascaris lumbricoides* IgG ELISA/EIA (catalog no. EIA-3817, lot ASCG-80; DRG Instruments GmbH, Marburg, Germany). This number was selected as a practical target for the secondary laboratory evaluation rather than through a separate sample-size calculation. Equal allocation of 100 specimens per region ensured that each region contributed equally to the comparison despite differences in the original cohort sizes.

Specimens were selected by purposive stratification according to region and the initial MVA GROUP result. The panel comprised 150 MVA GROUP-positive and 150 MVA GROUP-non-positive specimens, covering clearly negative, near-cutoff, weakly positive, and clearly positive reactivity. This composition allowed agreement to be examined across both result categories and around the assay cutoff. The selected panel was intended for assay comparison, not for estimating regional seroprevalence.

The selected specimens were tested according to the DRG instructions. Serum diluted 1:101 was incubated in antigen-coated wells for 60 min at 37 °C. After three washes with the supplied buffer, peroxidase-labeled Protein A was incubated for 30 min at room temperature, followed by three further washes. Tetramethylbenzidine development lasted 15 min at room temperature, and absorbance was measured at 450/620 nm. Results were expressed in DRG units (DU), with >11 DU positive, 9–11 DU equivocal, and <9 DU negative [16]. All 300 specimens had definitive positive or negative DRG results; no equivocal DRG results were observed. Overall, positive, and negative percent agreement were calculated from the paired classifications, with DRG as the comparator. Cohen’s κ assessed agreement beyond chance, and the exact McNemar test assessed asymmetry of discordant pairs. DRG was a commercial comparator rather than an independent reference standard for active infection.

### 2.9. Statistical Analysis

Continuous variables were summarized as mean ± standard deviation and categorical variables as counts and percentages. Proportions used Wilson 95% confidence intervals (CIs). Categorical comparisons used Pearson’s chi-square test, with Fisher’s exact test for 2 × 2 tables containing an expected count <5. Logistic regression estimated adjusted odds ratios (aORs) and Wald 95% CIs for seropositivity. The demographic model included region, age per 10 years, sex, residence, and sampling year. Each questionnaire variable was then added separately to this model. An interaction between reported consumption of unwashed vegetables and region was evaluated using a likelihood-ratio test with 2 degrees of freedom. Covariates were selected for their epidemiological relevance rather than univariable significance. Analyses used available complete records without imputation. Tests were two-sided, with *p* < 0.05; questionnaire analyses were exploratory and were not adjusted for multiple comparisons. Calculations used Python 3.12 and SciPy 1.17.

## 3. Results

### 3.1. Characteristics of the Study Population

The analytical sample comprised 2001 volunteers: 1001 from Akmola and 500 each from Pavlodar and North Kazakhstan. Women accounted for 1327 participants (66.3%), and the overall mean age was 47.2 ± 15.2 years. Mean age was highest in North Kazakhstan (51.5 ± 15.3 years), compared with Akmola (45.0 ± 14.7) and Pavlodar (47.2 ± 15.2). Urban residence was recorded for 1739 participants (86.9%). Rural residence was more common in North Kazakhstan (46.6%) than in Akmola (1.6%) or Pavlodar (2.0%). The demographic breakdown is provided in Table S1.

### 3.2. Regional Serological and Copromicroscopic Findings

Anti-*Ascaris* IgG was detected in 561/2001 participants (28.0%; 95% CI: 26.1–30.0%). Seroprevalence was 31.3% in Akmola (313/1001; 95% CI: 28.5–34.2%), 33.8% in Pavlodar (169/500; 95% CI: 29.8–38.1%), and 15.8% in North Kazakhstan (79/500; 95% CI: 12.9–19.3%). The overall regional difference was statistically significant (χ^2^ = 50.52, df = 2, *p* < 0.001), whereas Akmola and Pavlodar did not differ significantly in the unadjusted comparison (OR 1.12; 95% CI: 0.89–1.41; *p* = 0.322, Pavlodar vs. Akmola).

Kato–Katz examination detected compatible eggs in 169/2001 participants (8.4%; 95% CI: 7.3–9.7%): 81/1001 in Akmola (8.1%), 53/500 in Pavlodar (10.6%), and 35/500 in North Kazakhstan (7.0%). The regional difference in egg detection was not statistically significant (χ^2^ = 4.51, df = 2, *p* = 0.105).

### 3.3. Diagnostic Performance Relative to Kato–Katz

Most Kato–Katz-positive participants were ELISA positive. Discordance occurred predominantly among ELISA-positive participants without detectable eggs, resulting in a lower positive predictive value than sensitivity or negative predictive value relative to Kato–Katz (Table 1).

### 3.4. Demographic and Questionnaire Associations

Seropositivity was similar in women and men (28.2% vs. 27.7%; *p* = 0.836) and ranged from 25.5% to 29.7% across age groups in the pooled sample (*p* = 0.671; Table S1). After adjustment for age, sex, residence, and sampling year, the odds of seropositivity remained higher in Akmola (aOR 2.30; 95% CI: 1.59–3.33) and Pavlodar (aOR 2.37; 95% CI: 1.51–3.73) than in North Kazakhstan (both *p* < 0.001). The other demographic covariates were not statistically significant (Table S3A).

Cat, dog, and farm-animal ownership were reported by 806 (40.3%), 534 (26.7%), and 395 (19.7%) participants, respectively. Among 1977 participants with recorded dietary information, 121 (6.1%) reported consuming unwashed vegetables and 68 (3.4%) reported consuming raw meat. The crude inverse association with dog ownership (OR 0.69; 95% CI: 0.55–0.87) was absent after demographic and year adjustment (aOR 0.99; 95% CI: 0.76–1.29). No questionnaire variable showed a statistically significant pooled adjusted association (all *p* ≥ 0.097). Regional estimates for unwashed vegetables differed, whereas the pooled adjusted estimate was 1.44 (95% CI: 0.94–2.21; *p* = 0.097). Complete crude and adjusted comparisons are provided in Tables S2 and S3.

### 3.5. Agreement with the DRG ELISA

The two ELISAs showed high qualitative agreement in the selected serum panel. Discordant classifications were nearly evenly distributed between the two directions; the McNemar test did not identify a directional imbalance (Table 2).

The MVA GROUP and commercial DRG ELISAs demonstrated high qualitative agreement. Discordant classifications were distributed similarly in both directions, with no evidence of systematic directional disagreement between the assays (Table 2).

## 4. Discussion

This study extends our previous investigation in Almaty and the Almaty Region [11] by examining adult exposure to *Ascaris* across three additional regions. The regional serological pattern remained after adjustment for the measured demographic characteristics and sampling year, suggesting that these characteristics did not fully explain the differences between the recruited groups. National surveillance continues to document ascariasis in Kazakhstan [17], while the present survey adds information about antibody responses among adults recruited outside routine case detection. The comparison with stool examination also shows why regional seroprevalence and the prevalence of detectable intestinal egg shedding need separate interpretation.

The higher seropositivity in Akmola and Pavlodar compared with North Kazakhstan supports further investigation of local exposure conditions. Geographical variation has been documented within and between countries [18], and sanitation, food supply, and environmental conditions affecting egg survival may contribute to such differences [19]. However, North Kazakhstan had the largest proportion of rural participants and the lowest seropositivity in this survey. Rural residence alone therefore did not account for the observed pattern. Household sanitation, food handling, previous residence, and exposure outside the home may be more informative than an administrative urban–rural classification. These factors offer plausible directions for investigation, although the study did not establish the environmental causes of the regional differences.

The questionnaire provides useful context without identifying a single explanation for seropositivity. Ownership of cats, dogs, or farm animals does not directly measure exposure to infective eggs, and access to a garden gives little information about cultivation or hygiene practices. Water-source categories likewise do not describe treatment or storage. The disappearance of the crude inverse association with dog ownership after adjustment illustrates how participant characteristics can influence these comparisons. Contaminated vegetables remain a biologically plausible source of *Ascaris* exposure [20]. The Akmola association with reported consumption of unwashed vegetables was not reproduced in the other regions or established in the pooled adjusted analysis. Its interpretation is therefore exploratory: differences in food sourcing or preparation could contribute, but the small exposed group and differences in recording dietary responses could also influence the estimate. No local produce testing was available to corroborate a particular source.

The similar seropositivity across adult age groups and between women and men suggests that exposure assessment should extend across demographic groups. Comparable infection prevalence in men and women has also been reported internationally [21], although stool-based estimates cannot be directly compared with antibody prevalence. Current questionnaire responses may only partly capture exposures that preceded enrollment, whereas a qualitative IgG result also depends on the persistence and strength of the antibody response. More specific questions about the frequency and timing of food and household practices would help relate serological findings to potential exposures.

The raw-meat variable has relevance beyond conventional intestinal ascariasis. Humans can acquire *Toxocara* larvae by consuming undercooked tissues of infected paratenic hosts [22], and *A. suum* has also been investigated as a cause of visceral larva migrans using species-directed serological methods [23]. These observations explain the inclusion of dietary practices in a questionnaire developed for several parasitic infections. They also raise the possibility that exposure to related ascarids could affect antibody assays through shared antigen recognition. The present study did not demonstrate an association with raw-meat consumption or identify meat-borne infection, so this remains a biological consideration rather than an explanation assigned to individual participants.

The excess of antibody-positive over egg-positive findings is consistent with the different biological processes measured by ELISA and Kato–Katz. Antibodies may persist after earlier exposure [11], while *Ascaris* larvae undergo tissue migration before egg-producing adults develop [24]. Repeated exposure could also sustain antibody responses without a corresponding egg-positive result at enrollment. Larval infection that fails to progress to intestinal establishment is another possible explanation, although it was not assessed here. The host relationship is species-dependent: humans normally support the complete life cycle of *A. lumbricoides*, whereas *Toxocara* larvae remain in tissues without producing eggs [22,24]. Consequently, antibody positivity without eggs cannot by itself establish that a participant has a larval infection or is acting as a paratenic host. Low egg output and variation in antibody responses provide additional explanations for discordance in either direction.

Cross-reactivity should also be considered in relation to the antigens and detection reagents used. Shared recognition of *Toxocara* and *Ascaris* antigens has been demonstrated by immunoblotting [25]. In contrast, Mugo et al. observed little cross-reactivity with *Toxocara* when examining antibody responses to adult *Ascaris* excretory–secretory products [26]. This supports the value of antigen selection, while showing that findings from one assay cannot be transferred directly to another. The DRG instructions similarly acknowledge possible reactions with antibodies to other helminths [16]. Cross-reactivity is therefore one possible contributor to discordance, alongside infection stage, previous exposure, and the sensitivity of stool examination; its contribution was not quantified in this survey.

The diagnostic-performance estimates describe how the MVA GROUP assay classified participants relative to Kato–Katz detection of egg-shedding infection. Antibodies were detected in most participants with positive stool findings, supporting the assay’s ability to identify a response associated with established intestinal infection. However, the lower positive predictive value shows that a positive antibody result cannot be equated with current egg shedding. The high negative predictive value must also be considered in relation to the relatively low frequency of egg-positive findings in the sample. These observations support combining the two measurements in epidemiological work, with stool examination documenting egg shedding and serology adding information about the host response.

Agreement with DRG addresses the consistency of antibody classification and should be distinguished from performance relative to copromicroscopy. Both ELISAs use antigen-coated wells, peroxidase detection, and tetramethylbenzidine development. The MVA GROUP assay uses an anti-human IgG conjugate, whereas the DRG instructions specify peroxidase-labeled Protein A [11,16]. Serum dilution, incubation conditions, and cutoff calculation also differ. Recognition of overlapping antibody responses provides a plausible explanation for the high qualitative agreement despite these procedural differences, without requiring identical optical densities or antigen composition. The nearly balanced discordant classifications provide no evidence of a systematic tendency for one assay to classify more specimens as positive in this panel. Selection across negative, near-cutoff, and positive responses allowed this comparison over a range of antibody signals. Its estimates nevertheless describe the selected panel and do not independently establish specificity for current intestinal infection.

Pairing serum and stool findings with individual questionnaire data is a strength of the study because it allows regional patterns to be examined alongside both laboratory outcomes and reported exposures. The principal qualifications concern generalization and timing. Volunteer recruitment does not provide population-representative regional estimates, and self-reported practices may differ from those at the time of infection. Sampling year also overlapped only partly between regions, so adjustment based on repeated Akmola sampling could not fully separate geographical and temporal influences. These considerations define the scope of the comparisons while preserving their value as a basis for further regional research.

For future surveys, consistent recruitment, exposure questions, and laboratory procedures would help determine whether the regional serological pattern persists. The paired approach is also relevant to evaluating anthelmintic programs: stool findings can document changes in egg shedding, while serial antibody measurements could help characterize longer-term responses and renewed exposure. Research on antibodies to adult *Ascaris* excretory–secretory products supports further evaluation of this application [26]. The timing of antibody decline after treatment must first be established for the assay being used, because persistent IgG alone cannot demonstrate treatment failure or justify applying stool-prevalence thresholds to serology. Linking human surveys with investigations of food, water, and environmental contamination would strengthen the connection between exposure assessment and practical prevention, consistent with a One Health approach [19].

## 5. Conclusions

Anti-*Ascaris* IgG seropositivity was higher in the Akmola and Pavlodar volunteer samples than in North Kazakhstan, including after adjustment for measured demographic characteristics and sampling year. Agreement with DRG supports consistent antibody classification in the selected comparison panel. The comparison with Kato–Katz shows that antibody positivity and detectable intestinal egg shedding require separate interpretation. These findings support further regional studies combining exposure assessment with stool examination.

## Figures and Tables

**Table 1:** MVA GROUP ELISA performance relative to Kato–Katz copromicroscopy.

A. Paired results			
**MVA GROUP Result**	**Kato–Katz Positive, *n***	**Kato–Katz Negative, *n***	**Total, *n***
Positive	144	417	561
Non-positive	25	1415	1440
Total	169	1832	2001
B. Performance estimates			
**Measure**		**Estimate**	**95% CI**
Sensitivity		85.2%	79.1–89.8
Specificity		77.2%	75.3–79.1
Positive predictive value		25.7%	22.2–29.4
Negative predictive value		98.3%	97.4–98.8
Overall accuracy		77.9%	76.0–79.7
Cohen’s κ		0.304	—

Estimates concern ELISA classification relative to Kato–Katz detection of egg-shedding infection. Percentage CIs use the Wilson method. CI, confidence interval. —, a CI was not calculated for κ.

**Table 2:** Agreement between the MVA GROUP and DRG IgG ELISAs.

A. Paired results			
**MVA GROUP Result**	**DRG Positive, *n***	**DRG Negative, *n***	**Total, *n***
Positive	142	8	150
Non-positive	7	143	150
Total	149	151	300
B. Agreement estimates			
**Measure**		**Estimate**	**95% CI**
Positive percent agreement		95.3%	90.6–97.7
Negative percent agreement		94.7%	89.9–97.3
Overall agreement		95.0%	91.9–96.9
Cohen’s κ		0.900	—
Exact McNemar *p* value		1.000	—

DRG is the comparator for positive and negative percent agreement. Percentage CIs use the Wilson method. Estimates refer to the selected serum panel. CI, confidence interval. —, a CI was not calculated for κ and is not applicable to the McNemar *p* value.

## Data Availability

Data are contained within the article.

## Supplementary Materials

The following supporting information can be downloaded at https://www.mdpi.com/article/10.3390/life16101635/s1, Table S1, demographic characteristics and seropositivity by age and sex; Table S2, crude questionnaire associations by region and overall; Table S3, Adjusted associations with anti-Ascaris IgG seropositivity.
