# Supplementary material for: Anti-Ascaris IgG Seroprevalence and ELISA Performance Among Adults in Three Regions of Kazakhstan
Source: Life (Basel). 2026 Sep 30;16(10):1635. doi: 10.3390/life16101635 (PMC13705662; doi:10.3390/life16101635)
Supplement: Supplementary file 1 [file life-16-01635-s001.zip › life-4581198-supplementary.pdf]

Supplementary Materials

Anti-*Ascaris* IgG Seroprevalence and ELISA Performance among Adults in Three Regions of Kazakhstan

Manuscript life-4581198

Table S1. Demographic characteristics and seropositivity by age and sex.

A. Participant characteristics

Characteristic	Akmola<br>n = 1,001	Pavlodar<br>n = 500	North Kazakhstan<br>n = 500	Overall<br>N = 2,001
Participants	1,001	500	500	2,001
Mean age, years, mean ± SD	45.0 ± 14.7	47.2 ± 15.2	51.5 ± 15.3	47.2 ± 15.2
Urban residence	985 (98.4)	490 (98.0)	267 (53.4)	1,742 (87.1)
Rural residence	16 (1.6)	10 (2.0)	233 (46.6)	259 (12.9)

All participants were aged 18 years or older. Values are n (%) or mean ± standard deviation. Residence percentages use the full regional sample. Age and sex category totals are the denominators in Panel B.

B. Anti-Ascaris IgG seropositivity

Characteristic	Akmola	Pavlodar	North Kazakhstan	Overall
Women	188/594 (31.6)	129/373 (34.6)	57/360 (15.8)	374/1,327 (28.2)
Men	125/407 (30.7)	40/127 (31.5)	22/140 (15.7)	187/674 (27.7)
18–24 years	35/107 (32.7)	12/34 (35.3)	4/31 (12.9)	51/172 (29.7)
25–44 years	118/385 (30.6)	65/193 (33.7)	24/126 (19.0)	207/704 (29.4)
45–59 years	101/328 (30.8)	52/149 (34.9)	27/174 (15.5)	180/651 (27.6)
60–74 years	53/169 (31.4)	33/104 (31.7)	21/147 (14.3)	107/420 (25.5)
≥75 years	6/12 (50.0)	7/20 (35.0)	3/22 (13.6)	16/54 (29.6)

Panel B values are seropositive/tested (%). Age-group comparisons: Akmola, p = 0.705; Pavlodar, p = 0.988; North Kazakhstan, p = 0.820; overall, p = 0.671. Sex comparisons: Akmola, p = 0.753; Pavlodar, p = 0.525; North Kazakhstan, p = 0.974; overall, p = 0.836.

Table S2. Crude questionnaire associations by region and overall.

Each cell shows seropositive/total participants reporting versus not reporting the characteristic, followed by the crude OR (95% CI) and p value.

Reported characteristic	Akmola	Pavlodar	North Kazakhstan	Overall
Cat ownership	131/382 vs. 182/619<br>1.25 (0.95–1.65)<br>p = 0.105	38/135 vs. 131/365<br>0.70 (0.45–1.08)<br>p = 0.104	46/289 vs. 33/211<br>1.02 (0.63–1.66)<br>p = 0.933	215/806 vs. 346/1195<br>0.89 (0.73–1.09)<br>p = 0.266
Dog ownership	66/216 vs. 247/785<br>0.96 (0.69–1.33)<br>p = 0.798	15/47 vs. 154/453<br>0.91 (0.48–1.73)<br>p = 0.774	41/271 vs. 38/229<br>0.90 (0.55–1.45)<br>p = 0.655	122/534 vs. 439/1467<br>0.69 (0.55–0.87)<br>p = 0.002
Farm-animal ownership	64/188 vs. 249/813<br>1.17 (0.84–1.64)<br>p = 0.363	7/26 vs. 162/474<br>0.71 (0.29–1.72)<br>p = 0.446	29/181 vs. 50/319<br>1.03 (0.62–1.69)<br>p = 0.918	100/395 vs. 461/1606<br>0.84 (0.65–1.08)<br>p = 0.179
Garden or vegetable-garden access	7/19 vs. 306/982<br>1.29 (0.50–3.31)<br>p = 0.597	18/54 vs. 151/446<br>0.98 (0.54–1.78)<br>p = 0.939	1/8 vs. 78/492<br>0.76 (0.09–6.25)<br>p = 1.000†	26/81 vs. 535/1920<br>1.22 (0.76–1.97)<br>p = 0.406
Tap-water use	108/346 vs. 205/655	90/246 vs. 79/254<br>1.28 (0.88–1.85)<br>p = 0.195	60/375 vs. 19/125<br>1.06 (0.61–1.86)<br>p = 0.832	258/967 vs. 303/1034

	1.00 (0.75–1.32) p = 0.978			0.88 (0.72–1.07) p = 0.192
Use of open-water sources	48/144 vs. 265/857 1.12 (0.77–1.63) p = 0.564	13/43 vs. 156/457 0.84 (0.42–1.65) p = 0.605	24/151 vs. 55/349 1.01 (0.60–1.70) p = 0.970	85/338 vs. 476/1663 0.84 (0.64–1.10) p = 0.195
Consumption of unwashed vegetables	14/17 vs. 297/970 10.57 (3.02–37.07) p < 0.001	10/32 vs. 156/458 0.88 (0.41–1.90) p = 0.745	11/72 vs. 68/428 0.95 (0.48–1.91) p = 0.896	35/121 vs. 521/1856 1.04 (0.69–1.56) p = 0.839
Consumption of raw meat	6/16 vs. 305/971 1.31 (0.47–3.64) p = 0.603	3/12 vs. 163/478 0.64 (0.17–2.41) p = 0.759†	8/40 vs. 71/460 1.37 (0.61–3.09) p = 0.448	17/68 vs. 539/1909 0.85 (0.48–1.48) p = 0.560

Animal categories overlap. Non-reporting is the reference. Blank dietary responses were excluded: Akmola n = 987, Pavlodar n = 490, North Kazakhstan n = 500, and overall n = 1,977. Other comparisons use the full regional samples. OR, odds ratio; CI, confidence interval. OR intervals use the log-Wald method. Fisher's exact test; other p values use Pearson's chi-square test without continuity correction. Analyses are exploratory without adjustment for multiple comparisons.

Table S3. Adjusted associations with anti-*Ascaris* IgG seropositivity.

A. Regional and demographic characteristics

**Characteristic**	**aOR (95% CI)**	**p value**
Akmola vs. North Kazakhstan	2.30 (1.59–3.33)	<0.001
Pavlodar vs. North Kazakhstan	2.37 (1.51–3.73)	<0.001
Age, per 10-year increase	0.99 (0.92–1.05)	0.696
Women vs. men	1.05 (0.85–1.30)	0.663
Rural vs. urban residence	1.08 (0.71–1.63)	0.731
2026 vs. 2025 sampling	0.85 (0.65–1.11)	0.234

Panel A presents a single model adjusted for all listed characteristics (n = 2,001).

B. Questionnaire variables: pooled adjusted estimates

**Reported characteristic**	**Model N**	**aOR (95% CI)**	**p value**
Cat ownership	2,001	1.08 (0.87–1.34)	0.473
Dog ownership	2,001	0.98 (0.75–1.28)	0.896
Farm-animal ownership	2,001	1.17 (0.87–1.56)	0.300
Garden or vegetable-garden access	2,001	1.04 (0.63–1.69)	0.890
Tap-water use	2,001	1.11 (0.90–1.37)	0.333
Use of open-water sources	2,001	1.08 (0.80–1.46)	0.598
Consumption of unwashed vegetables	1,977	1.44 (0.94–2.21)	0.098
Consumption of raw meat	1,977	1.19 (0.67–2.12)	0.558

Each questionnaire variable was added separately to the demographic model. Non-reporting is the reference. Pooled estimates assume a common association across regions.

C. Consumption of unwashed vegetables: regional interaction estimates

**Region**	**aOR (95% CI)**	**p value**
Akmola	9.99 (2.83–35.25)	<0.001
Pavlodar	0.88 (0.41–1.90)	0.745
North Kazakhstan	0.96 (0.48–1.92)	0.912

Panel C uses an exposure-by-region interaction model adjusted for age, sex, residence, and sampling year (n = 1,977). Interaction test: likelihood-ratio $\chi^2 = 15.34$, df = 2, $p < 0.001$. In Akmola, 17 participants reported this exposure, most in 2025; dietary recording formats differed across regions.

aOR, adjusted odds ratio; CI, confidence interval. CIs use the Wald method. Missing dietary responses were excluded from the corresponding models. All serological results were analyzed as positive or negative. No stepwise selection or adjustment for multiple comparisons was used.
